# Estimation of Cough Peak Flow Using Cough Sounds

**DOI:** 10.3390/s18072381

**Published:** 2018-07-22

**Authors:** Yasutaka Umayahara, Zu Soh, Kiyokazu Sekikawa, Toshihiro Kawae, Akira Otsuka, Toshio Tsuji

**Affiliations:** 1 Department of System Cybernetics, Institute of Engineering, Hiroshima University, 1-4-1 Kagamiyama, Higashi-Hiroshima, Hiroshima 739-8527, Japan; sozu@bsys.hiroshima-u.ac.jp; 2 Department of Rehabilitation, Faculty of Health Sciences, Hiroshima Cosmopolitan University, Hiroshima 731-3166, Japan; otsuka-1949@hcu.ac.jp; 3 Division of Physical Analysis and Therapeutic Sciences, Institute of Biomedical and Health Sciences, Hiroshima University, Hiroshima 734-8553, Japan; sekikawa@hiroshima-u.ac.jp; 4 Division of Rehabilitation, Department of Clinical Support, Hiroshima University Hospital, Hiroshima 734-8551, Japan; toshihiro@hiroshima-u.ac.jp

**Keywords:** cough sound, cough peak flow, microphone, cough ability, cough strength, in-ear microphone, smartphone

## Abstract

Cough peak flow (CPF) is a measurement for evaluating the risk of cough dysfunction and can be measured using various devices, such as spirometers. However, complex device setup and the face mask required to be firmly attached to the mouth impose burdens on both patients and their caregivers. Therefore, this study develops a novel cough strength evaluation method using cough sounds. This paper presents an exponential model to estimate *CPF* from the cough peak sound pressure level (*CPSL*). We investigated the relationship between cough sounds and cough flows and the effects of a measurement condition of cough sound, microphone type and participant’s height and gender on *CPF* estimation accuracy. The results confirmed that the proposed model estimated *CPF* with a high accuracy. The absolute error between *CPF*s and estimated *CPF*s were significantly lower when the microphone distance from the participant’s mouth was within 30 cm than when the distance exceeded 30 cm. Analysis of the model parameters showed that the estimation accuracy was not affected by participant’s height or gender. These results indicate that the proposed model has the potential to improve the feasibility of measuring and assessing *CPF*.

## 1. Introduction

Cough peak flow (CPF) is a measurement commonly used to evaluate the cough strength, which reflects the ability to expel airway secretions [1,2,3,4,5,6,7] and can predict the extubation [7,8] and reintubation outcomes [9,10,11]. Values of CPF below 160 L/min have been associated with infective airway clearance [6,7,12] and patients who can generate a CPF of more than 270 L/min have little risk of developing respiratory failure during upper respiratory tract infections [13]. In the previous studies, CPF was measured with various devices, such as flow meters, spirometers and pneumotachographs. However, not all medical facilities provide these devices [14]. Moreover, the complex device setup, including firmly attaching the facemasks and infection control filters on the patient [1], imposes burdens on both patients and their caregivers. In addition, the measured CPF value can vary depending on the type of facemask and filter.

Therefore, we propose a novel simple evaluation system for evaluating cough ability using cough sounds without the use of the facemask or the filter. Several previous studies have proposed methods to monitor cough frequency using a microphone [15,16,17,18,19,20,21,22] but not to monitor cough ability. If the assessment of the cough ability by cough sounds is feasible, it can be applied to patients in whom obtaining cough peak flow measurements using the current method is difficult. However, the relationship between cough flow and cough sounds has not yet been clarified.

This study tested the hypothesis that cough sounds are associated with cough flow and we propose a cough flow prediction model using cough sounds based on our previous work [23]. Experiments were performed to determine the optimal cough sound measurement method and to investigate the influence of microphone type and participant height and gender on the accuracy of the estimated *CPF* via cough sounds (*CPS*) in young healthy participants. The effectiveness of the proposed model was also verified by comparison with polynomial functions.

## 2. Materials and Methods

### 2.1. Ethics Statement

This study was conducted in accordance with the amended Declaration of Helsinki. The Hiroshima Cosmopolitan University Institutional Review Board (No. 2015031) approved the protocol and written informed consent for study participation and publication of identifying information/images in an online open-access publication was obtained from all participants.

### 2.2. Participants

A total of 73 young healthy participants who were screened for the absence of pulmonary illness (forced expiratory volume in 1 s of at least 70% of predicted) with no history of pulmonary disease were included in this study. The participants were non-smokers, were not taking any long-term prescription medications and did not have any other medical illnesses. The mean ± standard deviation age of the participants was 21.0 ± 1.2 years (range, 20 to 28 years). Table 1 shows the baseline characteristics of the participants. It should be noted that the mean values (standard deviations) of body weight and BMI in Japanese over 20 years old are 66.8 (10.6) kg and 23.7 (3.2)% in male and 53.2 (8.7) kg and 22.4 (3.4)% in female, respectively [24], which are almost the same as those of participants in experiment 1.

### 2.3. Cough Flow Measurements

**Figure 1:**
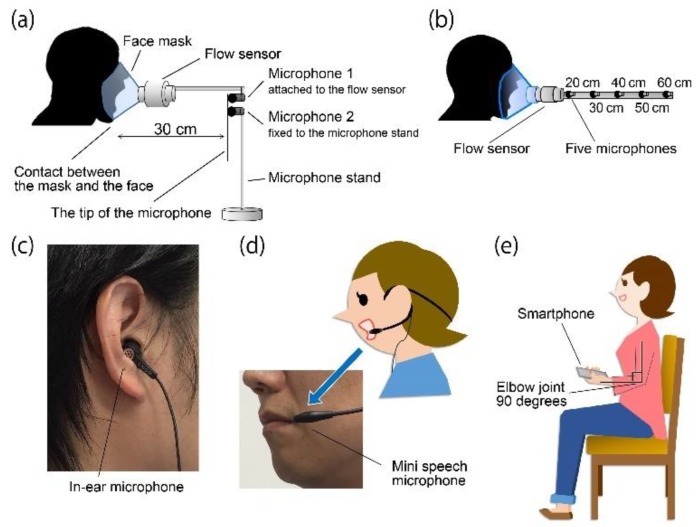
Experimental methods. (**a**) Experiment 1 method. The cough flow measurement is performed with the participants in a sitting position. The participants wear a face mask with an attached flow sensor. Two microphones are installed 30 cm from the point of face mask contact with the face. Microphone 1 is attached to the flow sensor and microphone 2 is fixed to the microphone stand; (**b**) Experiment 2 method. Microphones are installed 20 cm, 30 cm, 40 cm, 50 cm and 60 cm away from the point of face mask contact with the face; (**c**) In-ear microphone. The in-ear microphone was used in experiment 3 and fixed at the right external auditory canal; (**d**) Mini speech microphone. The mini speech microphone was used in experiment 3; (**e**) Smartphone microphone: The smartphone microphone was used in experiment 3.

Figure 1a shows the cough flow measurement method that was performed with the participants in a sitting position for all experiments. Participants wore a face mask with the flow sensor (Mobile Aeromonitor AE-100i; Minato Medical Science Co., Ltd., Osaka, Japan) attached. The measurement range of the flow sensor was 0–840 L/min and the measurement accuracy was within 3% of the indicated value. The cough flow signal was digitized using a 16-bit analogue-to-digital converter (PowerLab 16/35, ADInstruments, Inc., Dunedin, New Zealand) at a 100-kHz sampling rate set by analytical software (LabChart version 8, ADInstruments, Inc., Bella Vista, Australia). *CPF* was calculated from the maximal value of the cough flow data obtained under the different experimental conditions.

### 2.4. Cough Sound Measurements

Figure 1 shows the cough sound measurement method that was performed with the participants in a sitting position for all experiments.

*Experiment 1*. Cough sounds were measured using two microphones (AT9903, Audio-Technica Corporation, Tokyo, Japan), simultaneously with cough flow. The sensitivity of the microphones was −42 dB (0 dB = 1 V/1 Pa, 1 kHz). Both microphones were installed 30 cm away from the point of face mask contact with the face but by different installation methods to test their efficacy; that is, microphone 1 was fixed to the flow sensor [25] and microphone 2 was fixed to the microphone stand, as shown in Figure 1a.

*Experiment 2*. Cough sounds were simultaneously measured using five microphones of the same type as in experiment 1. Each microphone was installed at 20 cm, 30 cm, 40 cm, 50 cm and 60 cm away from the point of face mask contact with the face (Figure 1b).

**Figure 2:**
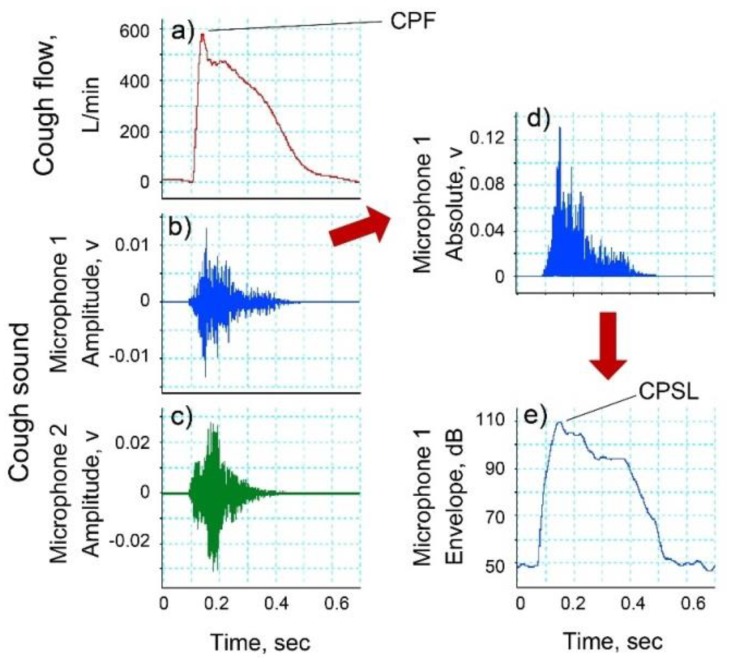
Examples of cough flow and cough sounds measured by microphones 1 and 2. (**a**) Experimental data of cough flow signals in experiment 1. *CPF*, cough peak flow; (**b**) Experimental data of cough sound signals measured by microphone 1 attached to the flow sensor in experiment 1. (**c**) Experimental data of cough sound signals measured by microphone 2 fixed to the microphone stand in experiment 1; (**d**) Absolute values of cough sound measured by microphone 1; (**e**) Envelope of cough sound signals calculated from the absolute cough sound values measured by microphone 1. The cough peak sound pressure level (*CPSL*) is defined as the maximum value of the envelope.

*Experiment 3.* Cough sounds were measured using three types of microphones. To measure cough sounds via from the right external auditory canal, an electret condenser microphone (ECM-TL3; Sony Corporation, Tokyo, Japan) (in-ear microphone) was attached to the right ear canal (Figure 1c). The sensitivity of the microphone was −35.0 dB (0 dB = 1 V/1 Pa, 1 kHz). A headset mini speech microphone (ECM-322BMP; Sony Corporation, Tokyo, Japan) (mini speech microphone) was attached to the left ear (Figure 1d). The sensitivity of the microphone was −42.0 dB (0 dB = 1 V/1 Pa, 1 kHz). The smartphone (iPhone 6 A1586; Apple, Inc., Cupertino, CA, USA) (smartphone microphone) was held in the left hand while the participant bent their elbow to 90° and their shoulder to 0° and then rotated their arm internally to 45° (Figure 1e). In the same manner as the cough flow method, analogue cough sound data were converted into digital signals at a sampling frequency of 100 kHz and stored on a personal computer. The digitized cough sound signals were bandpass filtered between 140 to 2000 Hz to minimize artifacts caused by heart sounds and muscle interference. Cough sound signals were converted into absolute values. Subsequently, the absolute values of the waveform of cough sounds were smoothed using a 20 ms time window to extract the envelopes [26]. The *CPSL* value was calculated from the maximal value of the cough sound data obtained under the different experimental conditions using LabChart version 8 software (Figure 2b,d,e).

### 2.5. Experimental Protocols

*Experiments 1 and 2*. Participants’ voluntary coughs were measured under three different conditions: five times with maximal effort from a maximal inspiratory level; five times with maximal effort from a resting inspiratory level; and five times with slight effort from a resting inspiratory level. Thus, a total of 15 cough flows and cough sounds per participant were measured simultaneously. Therefore, the total cough sample size was 495.

The participants were provided with sufficient instructions regarding the cough methods and practiced coughing in advance of the experiment. During the practice and experimental sessions, visual feedback of the flow-volume loop associated with inspiration and cough was provided in real time on a personal computer screen. In the measurement of slight cough, a physical therapist confirmed that the minimum effort cough was performed with proper cough sounds. The participants were allowed enough rest time between each trial to reduce the effects of fatigue.

*Experiment 3*. After providing sufficient instructions regarding the cough method to the participants, maximal voluntary coughing was performed three times. The participants had enough rest between each trial to reduce the effects of fatigue. *CPF* and *CPSL* were determined as the maximum value of each set of measured values.

### 2.6. Statistical Analysis

The relationship between *CPF* and cough sound was assumed to be in a form of exponential function, as in the following equation:

(1) $$CPF=\alpha \left(e^{\beta \cdot CPSL}-1\right),$$

where *α* and *β* are constants and *CPSL* is the maximum cough sound. Here, the maximum cough sound was used because previous studies have identified a correlation between the peak flow and the maximal absolute breath sound [27,28]. The Levenberg-Marquardt method was used to determine the coefficients in the proposed model; Equation (1) and the 95% CI of each coefficient, respectively. The Spearman’s rank correlation coefficient analysis was used to analyze the relationship between *CPS* and *CPF* and the estimation accuracy of *CPS*. In addition, the absolute error was calculated from *CPS* and *CPF*. Absolute reliability was investigated using the Bland-Altman analysis method to detect systematic bias, such as fixed and proportional bias. The Friedman test was used to compare the absolute error of the different distances from the sound source to the microphone and the different microphone types. The Mann-Whitney U test was used to compare the absolute error between the gender groups. The Bonferroni test was used for the post hoc analysis.

*The effect of participant’s height on the proposed model*. We hypothesized that coefficient α proportionally increases with height and we represented *α* using a linear term related to height, as in the following equation:

(2) $$CPF=\left(\alpha_{1}\cdot h+\alpha_{2}\right)\left(e^{\beta \cdot CPSL}-1\right),$$

where *h* represents participant’s height and *α*_1_, *α*_2_ and *β* are constants. The data from microphone 1 used in experiment 1 were also used for this experiment.

*The effect of participant’s gender on the proposed model*. To determine the effect of gender on *CPS*, we divided participants into male and female groups and coefficients *α* and *β* were calculated for each group using the Levenberg-Marquardt method. Moreover, Mann-Whitney’s U test was used to compare the coefficients between the two groups. The data recorded from microphone 1 in experiment 1 were used for this analysis. Finally, we verified the efficacy of the proposed model by comparison with first- to fourth-order polynomial functions, as in the following equation:

(3) $$CPF=\alpha_{3}\cdot CPSL,$$

(4) $$CPF=\alpha_{4}\cdot CPSL^{2}+\alpha_{5}\cdot CPSL,$$

(5) $$CPF=\alpha_{6}\cdot CPSL^{3}+\alpha_{7}\cdot CPSL^{2}+\alpha_{8}\cdot CPSL,$$

(6) $$CPF=\alpha_{9}\cdot CPSL^{4}+\alpha_{10}\cdot CPSL^{3}+\alpha_{11}\cdot CPSL^{2}+\alpha_{12}\cdot CPSL,$$

where *α*_3__–12_ are constants. The Levenberg-Marquardt method was also used to determine the coefficient in the equations and the coefficient of determination and the 95% CIs were calculated. The data from microphone 1 in experiment 1 were used for this analysis.

All statistical tests in this paper assumed a significance level of 0.05 and analyses were performed using G*power (version 3.1.9.2; University Kiel, Kiel, Germany) and SPSS Statistics (version 24.0; IBM, Chicago, IL, USA).

## 3. Results

### 3.1. Experiment 1: Relationships between CPF and CPSL and Verification of the Microphone Installation Method

**Figure 3:**
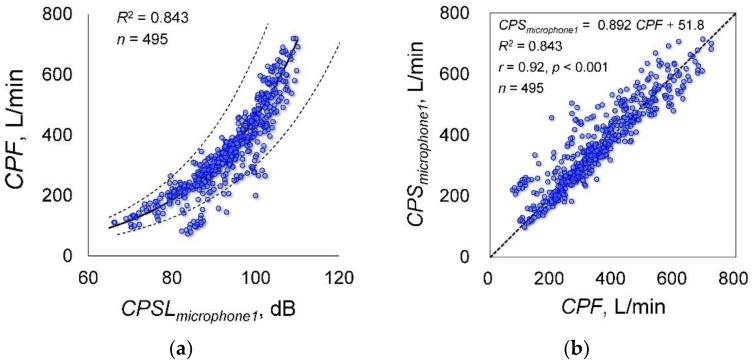
Estimation accuracy of Equation (7) calculated from the experimental data measured by microphone 1 attached to the flow sensor. (**a**) Relationship of *CPF* and *CPSL_microphone1_*. The solid lines represent the regression curves derived by fitting the coefficients in the proposed model using the Levenberg-Marquardt method based on *CPF* and *CPSL_microphone1_*. The dotted lines represent 95% confidence bands. *CPF*, cough peak flow; *CPSL_microphone1_*, cough peak sound pressure level by microphone 1; (**b**) Relationship between *CPF* and *CPS_microphone1_*. *CPS_microphone1_*, estimated cough peak flow calculated from *CPSL_microphone1_*.

Figure 2 shows examples of cough flow and cough sounds measured by microphones 1 and 2 (Figure 1a). Although the cough flow and cough sounds were measured using different methods, both responded to the initiation of the participant cough almost simultaneously. Figure 3a shows the relationship between *CPF* and *CPSL* measured by microphone 1 attached to the flow sensor. The coefficients of Equation (1), determined by the Levenberg-Marquardt method, are as follows: *α* = 5.67 (95% confidence internal (CI): 4.557 to 6.784) and *β* = 0.044 (95% CI: 0.042 to 0.046); the determination coefficient was 0.843. Therefore, the following estimation formula could be derived from *CPSL* measured by microphone 1:

(7) $$CPF=5.67\left(e^{0.044CPSL}-1\right).$$

Figure 3b shows the relationship between *CPF* and *CPS*, which confirmed a significant positive correlation (*r* = 0.920; *p* < 0.001; power, 100%).

In case of the experimental data measured by microphone 2 fixed to the microphone stand (Figure 1a), the Levenberg-Marquardt method showed that the coefficients obtained are as follows: *α* = 38.731 (95% CI: 24.071 to 53.391), *β* = 0.026 (95% CI: 0.023 to 0.030); the determination coefficient was 0.455. The Spearman’s rank correlation coefficient analysis showed a significant positive correlation between *CPF* and *CPS* (*r* = 0.699; *p* < 0.001; power, 100%). The Bland-Altman plot of *CPF* and *CPS* did not show fixed bias but did show proportional bias (*r* = −0.453; *p* < 0.001; power, 100%). Thus, the estimation accuracy of Equation (7) calculated from the experimental data measured by microphone 1 was higher than that measured by microphone 2.

### 3.2. Experiment 2: Effects of Microphone Distance from the Sound Source on Estimation Accuracy

**Figure 4:**
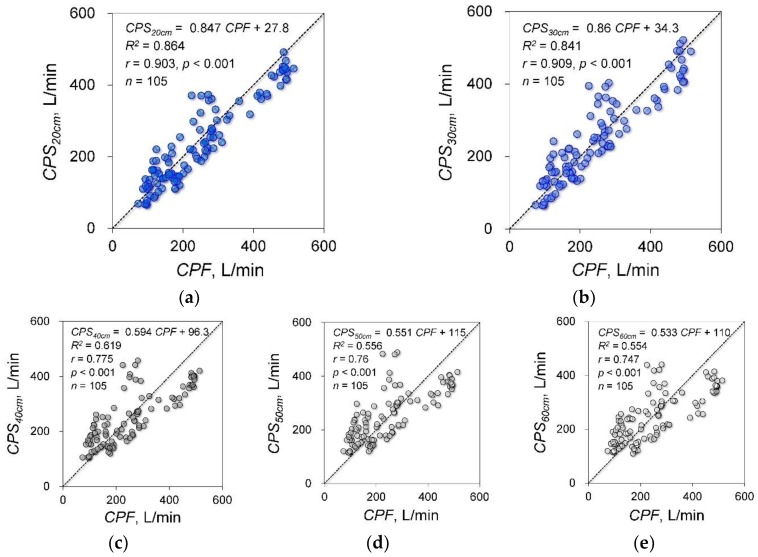
Estimation accuracy in each measurement condition. (**a**–**e**): Scatter plots of the measured data and the regression results of the proposed model: *CPSL*_20 cm__–60 cm_, cough peak sound pressure level measured by each microphone installed 20 cm, 30 cm, 40 cm, 50 cm and 60 cm from the point of face mask contact with the face; *CPS*_20 cm__–60 cm_, estimated cough peak flow calculated by *CPSL*_20 cm__–60 cm_. Solid lines represent regression lines derived from *CPF* and *CPSL*. The variation around the identity line is visibly reduced in graphs (**a**,**b**).

**Figure 5:**
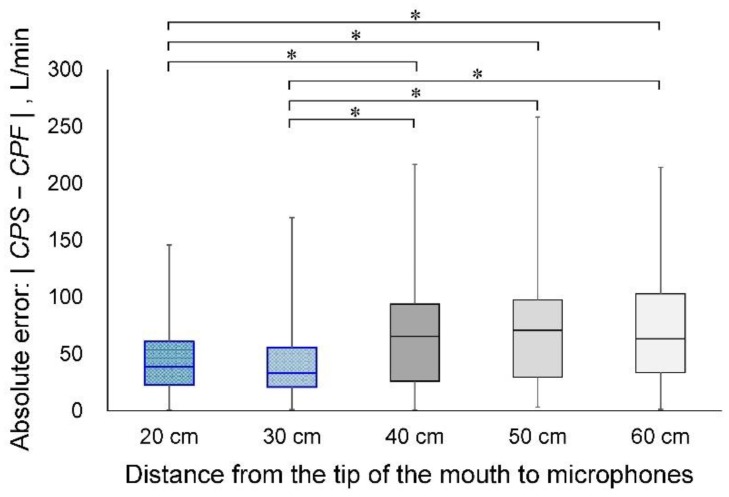
Comparison of the absolute error between each measurement condition. *CPF*, cough peak flow; *CPS*, estimated cough peak flow; * *p* < 0.001.

The cough sounds were measured by five microphones in the same model as experiment 1. These microphones were installed at 20 cm, 30 cm, 40 cm, 50 cm and 60 cm from the point of face mask contact with the face of the participant (Figure 1b). Figure 4 shows the results of the experimental data for each distance and the correlation analysis between *CPF* and *CPS*. The determination coefficients were 0.864 for 20 cm, 0.841 for 30 cm, 0.619 for 40 cm, 0.556 for 50 cm and 0.554 for 60 cm. The correlation coefficients were 0.903 (*p* < 0.001; power, 100%) for 20 cm, 0.909 (*p* < 0.001; power, 100%) for 30 cm, 0.775 (*p* < 0.001; power, 100%) for 40 cm, 0.76 (*p* < 0.001; power, 100%) for 50 cm and 0.747 (*p* < 0.001; power, 100%) for 60 cm. The absolute errors were 40.5 ± 26.7 L/min for 20 cm, 41.3 ± 30.6 L/min for 30 cm, 64.9 ± 46.2 L/min for 40 cm, 70.7 ± 49.0 L/min for 50 cm and 72.0 ± 47.5 L/min for 60 cm. Figure 5 shows the results of the Friedman and Bonferroni tests between the absolute error of each distance. The Friedman test showed that there was a significant difference among the absolute errors (*p* < 0.001). The Bonferroni test showed that the absolute errors were significantly lower for 20 cm and 30 cm than for 40 cm, 50 cm and 60 cm (*p* < 0.001).

### 3.3. Experiment 3: Effects of Microphone Type on Estimation Accuracy

**Figure 6:**
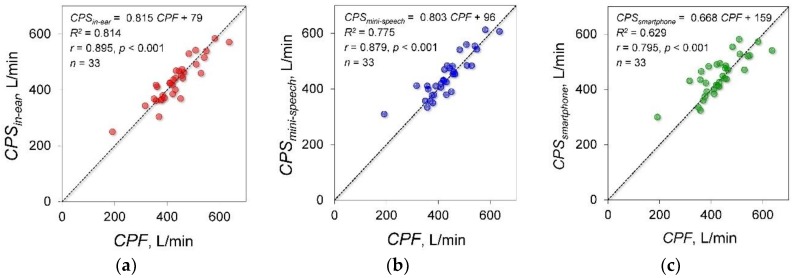
The results of experiment 3. *CPF*, cough peak flow; *CPS_in-ear_*, estimated *CPF* calculated from cough sound measured using the in-ear microphone; *CPS_mini-speech_*, estimated *CPF* calculated from cough sound measured using the mini speech microphone; *CPS_smartphone_*, estimated *CPF* calculated from cough sound measured using the smartphone microphone; (**a**) Relationship between *CPF* and *CPS_in-ear_*. (**b**) Relationship between *CPF* and *CPS_mini-speech_*. (**c**) Relationship between *CPF* and *CPS_smart_*.

A total of 33 young healthy participants were included in experiment 3. Based on the measurement of cough sounds using the in-ear microphone, the mini speech microphone and the smartphone microphone, *CPS_in-ear_*, *CPS_mini-speech_* and *CPS_smartphone_* were estimated, respectively. Figure 6 shows the results of the experimental data measured by each microphone and the correlation and regression analysis results between *CPF* and each *CPS*. The determination coefficients between *CPF* and each *CPS* estimated by the in-ear, mini speech and smartphone microphones were 0.763, 0.782 and 0.641, respectively. Significant positive correlations were found between *CPF* and each *CPS* (in-ear microphone: *r* = 0.895; *p* < 0.001; power, 100%, mini speech microphone: *r* = 0.879; *p* < 0.001; power, 100%, smartphone microphone: *r* = 0.795; *p* < 0.001; power, 99.9%). The absolute errors were 27.3 ± 22.6 L/min, 29.9 ± 27.4 L/min and 38.8 ± 35.7 L/min for the in-ear, mini speech and smartphone microphones. The Friedman test showed that there were no significant differences among the absolute errors (*p* = 0.157).

### 3.4. Effects of Participant’s Height on Estimation Accuracy

To consider the effect of height on the estimation accuracy of *CPF*, height parameters were introduced in the proposed model, such as Equation (2). The coefficients *a*_1_, *a*_2_ and *β* were determined by the Levenberg-Marquardt method using the measured data of experiment 1 measured by microphone 1 attached to the flow sensor. This model yielded a determination coefficient of 0.843. The determined coefficients were as follows: *a*_1_ = −0.001 (95% CI: −0.012 to 0.01), *a*_2_ = 5.767 (95% CI: 3.946 to 7.588) and *β* = 0.042 (95% CI, 0.04 to 0.044).

### 3.5. Effects of Gender on Estimation Accuracy

To consider the effect of gender on the estimation accuracy of *CPF*, the participants were divided into male and female groups and the coefficients of the proposed model, such as Equation (1), were determined for the respective groups. The coefficient *α* values were 8.3 ± 5.8 for the male group and 7.5 ± 5.4 for the female group. The Mann-Whitney U test showed no significant difference in the coefficient *α* values between the male and female groups (*p* = 0.653). The coefficient *β* values 0.049 ± 0.027 for the male group and 0.044 ± 0.01 for the female group. The Mann-Whitney U test showed no significant difference in the coefficient *β* values between the male and the female groups (*p* = 0.506).

### 3.6. Comparison between the Proposed Model and Polynomial Functions

**Figure 7:**
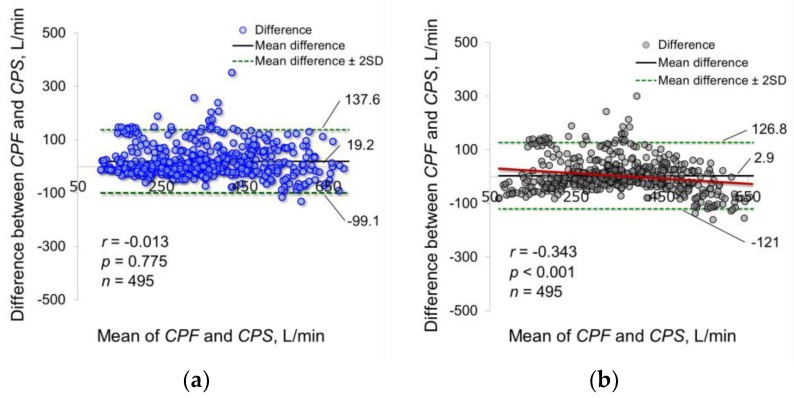
Bland-Altman plot of the measured and estimated cough peak flow. *CPF*, cough peak flow; *CPS*, estimated cough peak flow; (**a**) *CPS* estimated using our proposed model of Equation (1); (**b**) *CPS* estimated using Equation (4). Blue and black dots represent the difference between *CPF* and *CPS*. Bold black solid lines represent the mean difference between *CPF* and *CPS*. Green dotted lines represent the mean difference ± 2 standard deviation bands. Red lines represent the approximate straight line of the difference between *CPF* and *CPS* and the mean of *CPF* and *CPS*.

To demonstrate the efficacy of the proposed model, its estimation accuracy was compared with the polynomial functions without intercepts, such as Equations (3)–(6). The coefficients *α*_3__–12_ were determined by the Levenberg-Marquardt method in the same manner as in the proposed model. Table 2 shows the determined parameters and statistical analysis results. The 95% CIs of all coefficients in Equation (6) include 0, which indicates that the fourth-order polynomial function is redundant to estimate cough peak flow. Based on these results, the following analysis used our proposed model of Equation (1) and Equations (3)–(5). The absolute errors between *CPF* and *CPS* were 40.0 ± 41.8 L/min in the proposed model, 89.7 ± 65.2 L/min in Equation (3), 43.7 ± 39.9 L/min in Equation (4) and 98 ± 62.8 L/min in Equation (5). The Friedman test showed that there was a significant difference among the absolute errors (*p* < 0.001). The Bonferroni test showed that the absolute error was significantly lower in the proposed model and Equation (4) than in Equations (3) and (5) (*p* < 0.001). In addition, Figure 7 shows the corresponding Bland-Altman plot of the proposed model and Equation (4). The proposed model did not show fixed bias and proportional bias. However, Equations (4) did not show fixed bias but did show proportional bias (*r* = −0.343, *p* < 0.001).

## 4. Discussion

To the best of our knowledge, this is the first study to develop a method for estimating cough strength via cough sounds using a model represented by an exponential equation. Analysis of the results of experiment 1 demonstrated that *CPS* calculated from the cough sound measured using microphone 1 attached to a flow sensor is estimated to have high accuracy. Moreover, the *CPF* estimation accuracy using microphone 1 is significantly higher than that using microphone 2 fixed to the microphone stand. This is because microphone 1 was attached to the flow sensor, which maintained a fixed distance from the vocal cords but the distance between microphone 2, which was attached to the microphone stand and the vocal cords could be changed by inspiratory and/or coughing-induced body motion. The decrease in the sound level *L_p_* can be calculated by the distance from the sound source *r*_1_, *r*_2_ using the following equation:

(8) $$L_{p}=20\log\left(\frac{r_{2}}{r_{1}}\right).$$

The fact that the sound pressure level decreases with distance from the sound source indicates that body motion may be a cause of artefacts and, therefore, reduces estimation accuracy. Thus, to improve estimation accuracy, microphones should be installed on the body so that the microphones can maintain a constant distance from the sound source. Based on the results, we selected three types of wearable microphones (the in-ear microphone, the mini speech microphone and the smartphone microphone).

In experiment 2, we found that the distance between the mouth, as a sound source and the microphone needs to be less than 30 cm. Sound propagation in a room is a combination of direct and reflected sound waves from surfaces and boundaries in the room [29]. In addition, the sound attenuates with increasing distance from the sound source, as shown in Equation (8). When the microphone is set at a distance more than 30 cm from the mouth, the measured cough sounds may be influenced by sounds reflected from the walls and/or sound attenuation.

In experiment 3, we used three types of microphones (i.e., an in-ear microphone, a mini speech microphone and a smartphone microphone) to measure cough sounds. The strongest correlation between *CPF* and *CPS* was estimated using data obtained from the in-ear microphone. Thus, these microphones could be introduced as simple and wearable cough strength measurement devices.

In the respiratory function test, participant height is generally used to determine the normal level of respiratory function [30,31] and a relationship between *CPF* and height has been reported [32]. Based on these previous studies, we hypothesized that height affects *CPS*; however, in Equation (2), in which the coefficient *α* of Equation (1) was replaced by a linear function of height, the 95% CI of the multiplication coefficient *α*_1_ onto the height ranged from −0.112 to 0.02, including 0. This result indicates that height has a minimal effect on the *CPF* estimation accuracy.

Previous studies have also suggested that normal respiratory function levels vary according to gender [30,31] and the *CPF* of male participants has been shown to be significantly higher than that of female participants [32]. Therefore, we hypothesized that gender can affect the coefficients in the estimation equation; however, no significant difference in estimation accuracy was found between male and female participants. This result demonstrates that gender also has a minimal effect on the estimation accuracy when adopting the proposed model, represented by Equation (1).

We verified the prediction equation using first- to fourth-order polynomial functions. Several previous studies have reported that the relationship between air flow and breath sound amplitude is linear under high flow rate conditions [27,33]. It has also been reported that the sound amplitude during inspiration is proportional to the square of the air flow at the mouth [34,35]. Moreover, it was shown that the flow profile of inhalations can be estimated based on the logarithmic relationship between the acoustic envelope of the inhalation sound and the flow volume [28]. In contrast, a strong linear correlation between cough sound and cough flow has also been reported [36]. Our study revealed a nonlinear relationship between cough sound and cough flow. Thus, cough sounds were proportional to the square of the cough flow; however, a proportional bias was found in the second-order polynomial function, as observed for Equation (4). The effectiveness of the proposed model, represented by the exponential function, such as Equation (1), was verified by the fact that it successfully eliminated this systematic bias.

A major limitation of this study is that we did not fully consider the effect of age and disease, since the study participants were young Japanese healthy volunteers. In the respiratory function test, participant age is generally used to determine the normal level of respiratory function [30,30] and a relationship between *CPF* and age has been reported [32]. Also, the body weight or BMI of subjects may have impact on accuracy of estimated *CPF*. In addition, because the model was derived empirically in this study, its physiological and physical aspects must be addressed. Moreover, because the experimental results showed that maintaining a constant distance between the mouth, as a source of sound and the microphone is key to improving estimation accuracy, it will be necessary to develop a more suitable type of microphone. A wearable microphone, such as a piezoelectric bone conduction microphone, is one such candidate for application as the base technique.

## 5. Conclusions

This paper has proposed a nonlinear model for predicting the cough strength in young Japanese youth via cough sounds. Future studies should verify whether age, body weight and BMI influence the accuracy of the prediction model. If large variabilities in age, body weight and BMI that are to be expected in patients in the world are included and considered in the analysis, a practical device for assessing cough strength may be developed by employing the proposed model. The effects of age, body weight and BMI, the most suitable type of microphones and the physiological and physical explanations of the proposed model will be investigated in future work.

## Figures and Tables

**Table 1:** Characteristics of the participants.

Variable	Experiment 1 (*n* = 33)	Experiment 2 (*n* = 7)	Experiment 3 (*n* = 33)
Age, years	20.7 ± 1.0	22.0 ± 2.8	21.3 ± 0.4
Male gender, *n*	21	5	20
Height, cm	167 ± 7.9	167 ± 7.5	165 ± 8.4
Body weight, kg (male, female)	61.5 ± 12 (66.0 ± 12, 53.7 ± 5.4)	58.7 ± 9 (61.0 ± 7.6, 53.0 ± 11.3)	58.5 ± 11 (64.5 ± 11.3, 51.0 ± 6.2)
BMI, kg/m^2^ (male, female)	21.9 ± 3.2 (22.4 ± 3.6, 21 ± 2.1)	22.0 ± 2.8 (21.2 ± 2.0, 20.3 ± 2.2)	21.3 ± 0.5 (21.4 ± 0.6, 20.9 ± 0.5)

Values presented as means ± standard deviations.

**Table 2:** Relationship between cough peak flow and cough peak sound pressure level using Equations (2)–(4).

Estimation Equation	Coefficient	Estimated Value	Standard Error	95% CI		Determination Coefficient
				Lower	Upper	
Equation (3)	*α*_3_	3.819	0.053	3.714	3.923	0.373
Equation (4)	*α*_4_	0.117	0.003	0.111	0.124	0.822
	*α*_5_	−7.288	0.317	−7.910	−6.665	
Equation (5)	*α*_6_	0.002	0.000	0.002	0.003	0.843
	*α*_7_	−0.326	0.054	−0.431	−0.220	
	*α*_8_	13.013	2.2485	8.132	17.895	
Equation (6)	*α*_9_	0.019	0.000	−0.015	0.052	0.844
	*α*_10_	−0.005	0.007	−0.019	0.008	
	*α*_11_	0.355	0.617	−0.858	1.567	
	*α*_12_	−7.198	18.431	−43.412	29.016	
